# Molecular Regulatory Mechanism of the Iron-Ion-Promoted Asexual Sporulation of *Antrodia cinnamomea* in Submerged Fermentation Revealed by Comparative Transcriptomics

**DOI:** 10.3390/jof9020235

**Published:** 2023-02-10

**Authors:** Huaxiang Li, Jianing Dai, Yu Shi, Xiaoyan Zhu, Luqiang Jia, Zhenquan Yang

**Affiliations:** 1 College of Food Science and Engineering, Yangzhou University, Yangzhou 225009, China; 2 Jiangsu Provincial Key Construction Laboratory of Probiotics Preparation, Huaiyin Institute of Technology, Huaian 223003, China; 3 Jiangsu Key Laboratory of Dairy Biotechnology and Safety Control, Yangzhou University, Yangzhou 225009, China

**Keywords:** *Antrodia cinnamomea*, submerged fermentation, asexual sporulation, transcriptomics, metal ion, regulatory mechanism

## Abstract

*Antrodia cinnamomea* is a precious edible and medicinal fungus with activities of antitumor, antivirus, and immunoregulation. Fe^2+^ was found to promote the asexual sporulation of *A. cinnamomea* markedly, but the molecular regulatory mechanism of the effect is unclear. In the present study, comparative transcriptomics analysis using RNA sequencing (RNA-seq) and real time quantitative PCR (RT-qPCR) were conducted on *A. cinnamomea* mycelia cultured in the presence or absence of Fe^2+^ to reveal the molecular regulatory mechanisms underlying iron-ion-promoted asexual sporulation. The obtained mechanism is as follows: *A. cinnamomea* acquires iron ions through reductive iron assimilation (RIA) and siderophore-mediated iron assimilation (SIA). In RIA, ferrous iron ions are directly transported into cells by the high-affinity protein complex formed by a ferroxidase (FetC) and an Fe transporter permease (FtrA). In SIA, siderophores are secreted externally to chelate the iron in the extracellular environment. Then, the chelates are transported into cells through the siderophore channels (Sit1/MirB) on the cell membrane and hydrolyzed by a hydrolase (EstB) in the cell to release iron ions. The *O*-methyltransferase TpcA and the regulatory protein URBS1 promote the synthesis of siderophores. HapX and SreA respond to and maintain the balance of the intercellular concentration of iron ions. Furthermore, HapX and SreA promote the expression of *flbD* and *abaA*, respectively. In addition, iron ions promote the expression of relevant genes in the cell wall integrity signaling pathway, thereby accelerating the cell wall synthesis and maturation of spores. This study contributes to the rational adjustment and control of the sporulation of *A. cinnamomea* and thereby improves the efficiency of the preparation of inoculum for submerged fermentation.

## 1. Introduction

*Antrodia cinnamomea* (syn. *Antrodia camphorata*) is a precious edible and medicinal fungus that belongs to phylum Basidiomycetes, family Polyporaceae, and genus *Antrodia* [1]. *A. cinnamomea* presents various biological activities, such as hepatoprotective, antitumor, antioxidant, antiviral, antivasodilation, hypoglycemic, immunoregulatory, and gut microbiota regulatory [2,3,4,5]. The main bioactive compounds of *A. cinnamomea* include triterpenoids (such as antcins A, antcins B, antcins C, antcins H, antcins K, methylantcinate, and sulphurenic acid), polysaccharides, ubiquinone derivatives (such as antroquinonol, antroquinonol B, antroquinonol C, antroquinonol D, antroquinonol L, antroquinonol M, and 4-acetyantroquinonol B), maleic and succinic acid derivatives (such as antrodins A, antrodins B, antrodins C, antrodins D, and antrodins E), and benzene derivatives [4,6,7,8]. What is more, some compounds, such as antcins C, antcins K, antrodins B, antrodins C, and 4-acetyantroquinonol B, with excellent bioactivities from *A. cinnamomea*, have not been found in other edible and medicinal fungi so far [8,9].

*A. cinnamomea* has a huge market demand due to its outstanding biological activities and medicinal values. However, the wild fruiting bodies of *A. cinnamomea* are scarce and extremely expensive. Thus, the large-scale artificial culture of *A. cinnamomea* becomes necessary and important [10]. At present, the main techniques for the artificial culture of *A. cinnamomea* are basswood culture, plate culture, solid-state fermentation, and submerged fermentation [10,11]. Among them, the components of the fruiting bodies obtained from the basswood culture are the most similar to those of the wild fruiting bodies of *A. cinnamomea*, but the basswood must be from *Cinnamomum kanehirae Hay*, which is the only natural host of *A. cinnamomea* and is very expensive. In addition, the production periods of basswood culture are 1–5 years, which causes a high cost of time and materials. The production periods of plate culture are 2–4 months, and its yield of *A. cinnamomea* fruiting body is low, which cause low production efficiency by plate culture. The production periods of solid-state fermentation for *A. cinnamomea* are 1–2 months, and the *A. cinnamomea* mycelia obtained from solid-state fermentation cannot be separated from the culture medium, which cause a low content of the active compounds in the products of solid-state fermentation. Thus, the submerged fermentation becomes the most popular artificial culture method for *A. cinnamomea* because of the advantages of short fermentation period (10–14 days), high production efficiency, and easy to large-scale application [10,11,12].

Mycelium inoculation is usually used in the traditional submerged fermentation processes for *A. cinnamomea* [11,12], whereas mycelium inoculation in the submerged fermentation of *A. cinnamomea* presents some disadvantages, such as the difficult control of the quality and amount of inoculum (i.e., mycelia), and the poor synchronization of mycelial growth during fermentation, resulting in poor batch stability. Nevertheless, spore inoculation can solve these problems well [10]. However, the large-scale production of *A. cinnamomea* through submerged fermentation still has problems, such as the tedious and time-consuming preparation of inoculum (i.e., asexual spores) due to low sporulation, which severely limits the efficiency and benefit of *A. cinnamomea* production through submerged fermentation [11].

The sporulation of filamentous fungi is affected by various factors, such as nutrition, light, pH, and metal ions [13,14,15]. Among these factors, iron ions play an important role in the growth and development of most filamentous fungi [16,17]. For example, adding 56 µg/mL of iron could promote the growth and sporulation capacity of *Cylindrocarpon destructans* [18]. The growth rate and sporulation capacity of *Phanerochaete chrysosporium* could be significantly improved by the appropriate concentration of iron ions [19]. Iron balance is beneficial for promoting and enhancing the germination and infectivity of *Beauveria bassiana* spores [20].

Nonetheless, further studies on the molecular regulatory mechanisms of the iron-ion-promoted asexual sporulation of filamentous fungi remain lacking. What is more, there are no reports on iron ions promoting the asexual sporulation of *A. cinnamomea* in submerged fermentation and its mechanism so far. In the present study, comparative transcriptomics analysis using RNA-seq and RT-qPCR was conducted on *A. cinnamomea* mycelia cultured in the presence or absence of Fe^2+^. Then, the molecular regulatory mechanism underlying the Fe^2+^-promoted asexual sporulation of *A. cinnamomea* was revealed by combining relevant reports.

## 2. Materials and Methods

### 2.1. Strain

*A. cinnamomea* strains (No. ATCC 200183) were purchased from the American Type Culture Collection (Manassas, VA, USA).

### 2.2. Submerged Fermentation of A. cinnamomea

*A. cinnamomea* was cultured in accordance with the method reported by Li et al. [21]. In brief, the spore concentration (i.e., sporulation) of inoculum was counted and calculated using a hemocytometer under an optical microscope, then the *A. cinnamomea* was cultured in the fermentation medium (20.0 g/L glucose, 4.0 g/L yeast extract powder, 3.0 g/L KH_2_PO_4_, and 1.5 g/L MgSO_4_, with the initial pH of 4.5) at 26 °C and 150 r/min for 10–11 days, with an inoculum size of 1.0 × 10^6^ spores/mL.

### 2.3. Effects of Different Concentrations of Fe^2+^ on the Sporulation and Biomass of A. cinnamomea

FeCl_2_·4H_2_O was used to prepare the Fe^2+^ mother liquor, with a concentration of 1 mol/L. The corresponding volume of the Fe^2+^ mother liquor was added into the culture medium to adjust the Fe^2+^ concentrations of the culture medium to 0.05, 0.1, 0.2, 0.3, 0.4, and 0.5 mmol/L. Then, the culture medium was inoculated and cultured in accordance with the method described in Section 2.2. Samples were taken on days 6–11 to determine sporulation and biomass [22]. Medium with the same volume of deionized water added was regarded as the control group.

### 2.4. Sample Preparation for RNA-Seq

The fermentation broth of *A. cinnamomea* cultured in the presence or absence of Fe^2+^ was prepared in accordance with the method reported by Li et al. [23]. In brief, the *A. cinnamomea* mycelia pellets cultured in the presence or absence of Fe^2+^ (marked as “Fe” or “CK” group, respectively) for 7, 8, and 9 days were collected by filtering with four layers of gauze, then washed with Tris-EDTA (Ethylene Diamine Tetraacetic Acid) buffer solution (pH 8.0) and snap-frozen by liquid nitrogen. Three biological replicates were taken at each time point of each group.

### 2.5. RNA-Seq and Bioinformatics Analysis

The mycelial samples were sent to Beijing Novogene Biotechnology Co., Ltd. (Beijing, China) for high-throughput sequencing using an Illumina HiSeq™ 2500 (San Diego, CA, USA). The low-quality reads in the raw data were removed by FASTP software (version 0.19.7) with the parameters of “-g -q 5 -u 50 -n 15 -l 150” to obtain the qualified reads (i.e., clean reads). Then, the clean reads were used for de novo assembly by Trinity [24] software to obtain the transcript database. Finally, the unigene database (Supplementary Table S1) of *A. cinnamomea* was obtained after being compared against the genome database of *A. cinnamomea* (Accession number: GCA_000766995.1, NCBI).

The differentially expressed genes (DEGs) were obtained with the H-Cluster algorithm by Corset (https://code.google.com/p/corset-project/ accessed on 14 February 2022) [25]. The annotation and functional analysis of the DEGs were conducted using the NCBI database (https://www.ncbi.nlm.nih.gov/ accessed on 22 February 2022), GO database (http://geneontology.org/ accessed on 25 February 2022), and KEGG database (http://www.genome.jp/kegg/ accessed on 3 March 2022).

### 2.6. RT-qPCR Analysis

RT-qPCR analysis was performed in accordance with the reported method [23] using the same samples for RNA-seq and the 18S ribosomal ribonucleic acid (rRNA) sequence of *A. cinnamomea* as the internal reference. The *A. cinnamomea* mycelium samples cultured in the absence of Fe^2+^ were used as control. The RT-qPCR primer sequences of the related genes are shown in Table 1.

### 2.7. Statistical Analysis of Data

At least three replicates were prepared for each experimental group. Data were presented as mean ± standard deviation (SD). One-way analysis of variance (ANOVA) was carried out using SPSS PASW Statistics Version 18.0 (Palo Alto, CA, USA), and the significant difference level was set as *p* < 0.05. The principal component analysis (PCA) was performed based on the “Fragments Per Kilobase of exon model per Million mapped fragments” (FPKM) values of different groups with the “ggplot2” package of R (Version 3.0.3). For the differentially expressed gene analysis, it was considered as a significant difference when the fold change of genetic expression was more than 1.25 times (up-regulation, log2 fold change ≥ 0.32) or less than 0.80 times (down-regulation, log2 fold change ≤ −0.32).

## 3. Results and Discussion

### 3.1. Effects of Fe^2+^ on the Sporulation Capacity of A. cinnamomea

**Figure 1:**
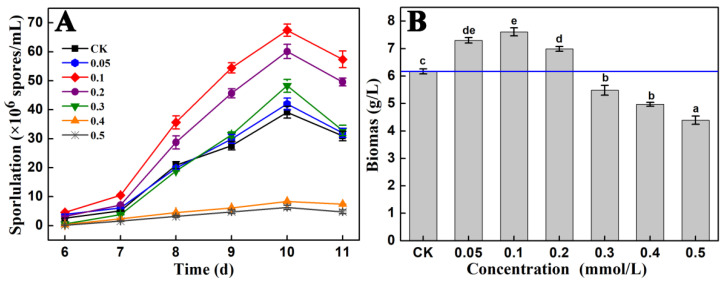
Effects of different concentrations of Fe^2+^ on the sporulation (**A**) and biomass (**B**) of *A. cinnamomea* in submerged fermentation. Note: “CK”, no metal ion was added in the culture medium; “0.05”, the concentration of Fe^2+^ in the culture medium is 0.05 mmol/L; “0.1, 0.2, 0.3, 0.4, and 0.5” are similar to “0.05 mmol/L Fe^2+^”. The *A. cinnamomea* mycelia cultured for 10 days at 26 °C and 150 r/min with inoculum size of 1.0 × 10^6^ spores/mL were used to measure the biomass; Different letters in (**B**) indicate significant differences at the level of 0.05 (*p* < 0.05).

As shown in Figure 1, the addition of 0.1–0.2 mmol/L Fe^2+^ markedly promoted the sporulation and growth of *A. cinnamomea* in submerged fermentation. The addition of 0.1 mmol/L Fe^2+^ had the most significant effect and increased the maximum sporulation of *A. cinnamomea* by 72.39%. However, when Fe^2+^ was added at concentrations exceeding 0.4 mmol/L, it had an obvious inhibitory effect on the growth of *A. cinnamomea* and dramatically inhibited sporulation. Therefore, Fe^2+^ can significantly promote the sporulation and growth of *A. cinnamomea* when its addition concentration is strictly controlled.

### 3.2. RNA-Seq and Statistical Analysis

#### 3.2.1. Preparation of Sequencing Samples

Figure 1 shows that *A. cinnamomea* started to produce spores rapidly on day 7. What is more, *A. cinnamomea* was in the early stage of rapid sporulation on day 8 and was in the middle stage of rapid sporulation on day 9. In other words, days 7, 8, and 9 were the beginning, early, and middle stages of the rapid sporulation of *A. cinnamomea* in submerged fermentation, respectively. Therefore, our sequencing samples were *A. cinnamomea* mycelia incubated for 7, 8, and 9 days in the culture medium without Fe^2+^ (referred to as “CK_7d”, “CK_8d”, and “CK_9d”, respectively) and *A. cinnamomea* mycelia incubated for 7, 8, and 9 days in the culture medium with 0.1 mmol/L Fe^2+^ (referred to as “Fe_7d”, “Fe_8d”, and “Fe_9d”, respectively) for a total of six groups. Three biological replicates were taken for each group. For example, three biological replicates of the “CK_7d” group were recorded as “CK_7d_1”, “CK_7d_2”, and “CK_7d_3”, and the rest may be deduced by analogy. A total of 18 samples were used for RNA-seq.

#### 3.2.2. Statistical Analysis of Sample Repeatability and DEGs

**Figure 2:**
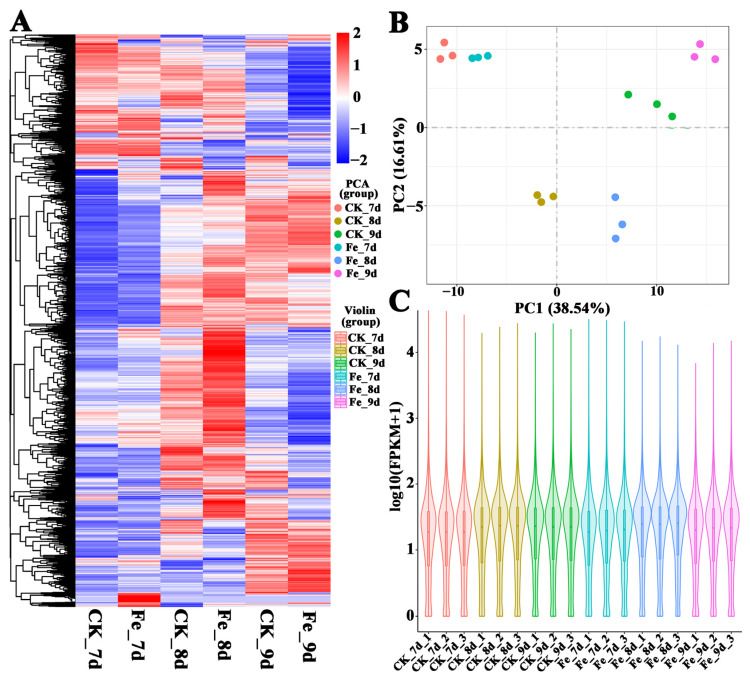
Statistical analysis of the RNA-seq samples. Note: (**A**): Cluster analysis of gene expression; (**B**): principal component (PC) analysis; (**C**): distribution of the FPKM values of genes in all samples.

On the basis of the expression of unigenes, which are represented by FPKM values (Supplementary Table S2), in various samples, we performed principal component analysis (PCA) (Figure 2A) and DEG analysis (Figure 2B) to investigate the consistency of the three biological repetitions and DEG distribution in the three sample groups.

From the statistical analysis, it was found that the three biological replicates in each group are concentrated, and the different groups can be clearly distinguished (Figure 2B), which indicated that the three biological replicates of each groups had good biological repeatability and the genes were expressed with significant differences between different groups. In addition, the distributions of the maximum, median, and minimum of all biological repetition samples were basically consistent, and gene expression was largely dispersed in different groups (Figure 2C), which verified the good biological repeatability of the replicates and significant differences between the groups again. What is more, it was shown that Fe^2+^ exerted a global impact on gene expression in *A. cinnamomea*, and the change in gene expression exhibited certain trends on the basis of incubation time and sample treatment (Figure 2A). In particular, some gene clusters were significantly up-regulated (color changed from blue to red) or significantly down-regulated (color changed from red to blue) compared with the control group after adding Fe^2+^ (Figure 2A), indicating that Fe^2+^ significantly affected the expression of some genes, and these gene are probably involved in iron ions promoting the asexual sporulation of *A. cinnamomea*. In sum, the results in Figure 2 indicate that the design of the sequencing sample is reasonable and that the sequencing quality is perfect.

#### 3.2.3. Enrichment Analysis of DEGs

**Figure 3:**
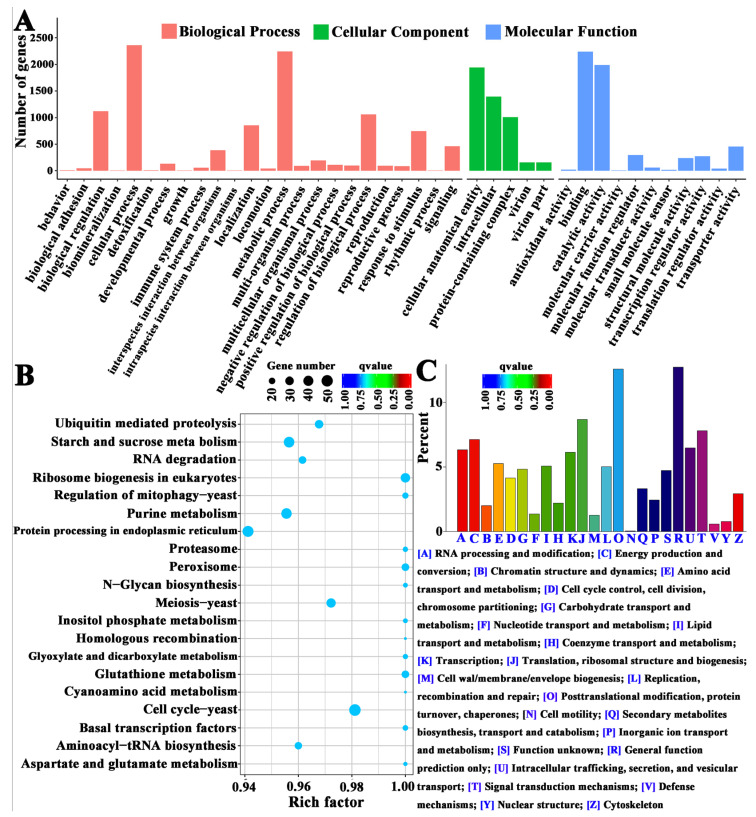
GO (**A**), KEGG (**B**), and KOG (**C**) enrichment analyses of DEGs.

The GO, KOG, and KEGG enrichment analyses of DEGs (Figure 3, Supplementary Table S3) revealed that the DEGs were involved in the cellular process, the developmental process, the reproductive process, transcription regulator activity, transporter activity, cell wall integrity (CWI), cell cycle control, cell division, signal transduction, amino acid metabolism, transport and catabolism, cell growth and death, sensory systems, and membrane transport. The CWI signaling pathway was reported to be closely related to cell wall synthesis and fungal sporulation in phytopathogenic fungi [26,27]. Serine metabolism is involved in the promotion of the sporulation of *Aspergillus fumigatus* by iron ions [28]. As inferred through functional classification, these genes may be related to the iron-ion-mediated asexual sporulation of *A. cinnamomea* and require further bioinformatics analysis.

### 3.3. Bioinformatic Analysis

First, on the basis of our previous study [21], we successfully obtained 18 genes related to the FluG-mediated asexual sporulation signaling pathway of *A. cinnamomea* from the unigene database, namely, *sfaD*, *fluG*, *velB*, *flbA*, *ganB*, *veA*, *vosA*, *fadA*, *flbC*, *pkaA*, *nsdD*, *flbD*, *wetA*, *abaA*, *flbB*, *stuA*, *brlA*, and *sfgA*. Subsequently, on the basis of the relevant references [26,28,29,30,31,32,33,34,35,36,37,38,39,40,41], we obtained 24 genes (Table 2) that may be related to the Fe^2+^-mediated asexual sporulation of *A. cinnamomea* among the DEGs (Supplementary Table S4), namely, *mirB*, *ftrA*, *hapX*, *sreA*, *fetC*, *bck1*, *uvt*, *urbs1*, *sit*, *fre*, *slt2*, *ssiG* 06045, *feoB*, *tpcA*, *nrps*, *nps2*, *nps4*, *clpP*, *mkk1*, *sidA*, *yvmB*, *fur*, *estB*, and *wsc1* (Table 2).

Iron, a trace element, is an indispensable factor for the growth of filamentous fungi. However, the molecular regulatory mechanism underlying the induction of the asexual sporulation of filamentous fungi by iron ions remains unclear. In *A. fumigatus*, reductive iron assimilation (RIA) includes the ferric reductase FreB, the ferroxidase FetC, and the iron permease FtrA [42,43]. Iron permease widely exists in organisms and belongs to the ferredoxin-dependent family [43]. Most fungal species, including siderophore producers or nonproducers, have at least one homologous iron permease gene [44].

Nonreductive iron assimilation works through the synthesis and utilization of siderophores. In *Aspergillus nidulans*, the deletion of the *sidA* gene results in the inability to synthesize siderophores [26]. Nonribosomal peptide synthetase (NRPS) is a multifunctional protein that can biosynthesize small peptides and is responsible for the synthesis of low-molecular-weight secondary metabolites. Among NRPSs, *nps2* is structurally conserved and functionally stable in many filamentous fungi and is closely related to the biosynthesis of extracellular and intracellular siderophores [29]. *nps4* plays an important role in the surface hydrophobicity of pathogenic bacteria and the formation of conidia [29]. In *A. fumigatus*, *mirb* transports the chelates of siderophores and Fe^2+^ into cells that are then hydrolyzed by hydrolase EstB for Fe^2+^ release [30,31]. Iron ion assimilation is thus achieved.

Fungi possess two transcriptional regulatory systems for jointly regulating iron homeostasis: the basic region-leucine zipper (bZIP)-type transcription factor *hap* and the GATA-type zinc finger transcription factor *sre* [32]. A negative feedback regulatory system is formed on the basis of these two factors to jointly regulate iron homeostasis [33]. The transcription factor *hapX* can regulate the iron acquisition and conidial infection ability of *Beauveria bassiana* in accordance with the concentration of iron ions [45]. HapX also affects the growth, sporulation, and conidial germination, and maintains iron homeostasis in fungi [46,47,48]. The zinc finger transcription factor *sre* is involved in the regulation of siderophore synthesis and iron assimilation in fungi. For example, the transcription factor *sreA* is involved in the transcriptional activation of genes related to the siderophore synthesis pathway in *A. fumigatus* [49]. SRE1 is involved in siderophore synthesis in *Histoplasma capsulatum* [34].

Moreover, the increase in biofilm synthesis by *A. fumigatus* induced by a low-iron condition is dependent on the CWI pathway because the receptor Wsc1 on the cell membrane in the CWI pathway is involved in the perception of biofilm synthesis [35,50]. In *B. bassiana, bck1*, *mkk1*, and *slt2* in the CWI pathway can not only maintain cell integrity but can also positively regulate growth, sporulation, and host infectivity [26,51]. The deletion of *yvmB* inhibits the sporulation of *Bacillus subtilis* and leads to the disruption of the proteins involved in iron transport [36]. The protein encoded by the *uvt3277* gene can transport iron ions [52]. In *Ustilago maydis*, the deletion of the *urbs1* gene inhibits siderophore biosynthesis and limits iron assimilation [41]. The *cmSIT1* gene is associated with siderophore-mediated transport [37].

### 3.4. RT-qPCR Analysis

**Figure 4:**
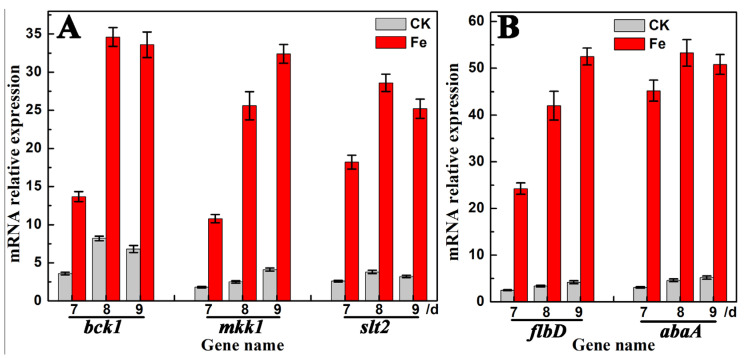
Expression levels of genes involve in CWI pathway (**A**) and FluG-mediated patthway (**B**) in the mycelium samples of *A. cinnamomea*. Note: “mRNA”, messenger RNA; “CK”, the mycelia cultured absence of Fe^2+^; “Fe”, the mycelia cultured in the presence of 0.1 mmol/L of Fe^2+^. The 18S rRNA gene of *A. cinnamomea* was taken as the internal reference.

By comparing the expression levels (FPKM values) of the genes shown in Table 2 in the sequencing samples, the *bck1*, *mkk1*, and *slt2* genes in the CWI pathway and the *flbD* and *abaA* genes in the FluG-mediated sporulation signaling pathway were found to exhibit a dramatic increment after Fe^2+^ treatment. Thus, RT-qPCR was adopted to further analyze and verify the expression patterns of these five genes in the samples. As shown in Figure 4, compared with those in the CK group, the expression levels of the *bck1*, *mkk1*, and *slt2* genes had increased by 3–18 times and the expression levels of the *flbD* and *abaA* genes had increased by 9–21 times in *A. cinnamomea* mycelia treated with Fe^2+^. Therefore, these five genes were speculated to play a key regulatory role in the iron-ion-promoted asexual sporulation of *A. cinnamomea*.

### 3.5. Model of the Signaling Pathway of the Iron-Ion-Promoted Asexual Sporulation of A. cinnamomea

In fungi, the iron ion is a cofactor of many enzymes. The regulation of the transmembrane assimilation of iron is a primary and important way to maintain intracellular iron homeostasis [28]. For this reason, fungi have evolved a variety of iron assimilation methods. These iron assimilation methods are generally divided into two categories: high-affinity iron assimilation and low-affinity iron assimilation [28]. The high-affinity iron assimilation method is divided into two types: RIA and siderophore-mediated iron assimilation (SIA) [53].

In *A. fumigatus*, iron assimilation through RIA first involves the reduction of trivalent iron ions into divalent iron ions with improved solubility under the action of ferric reductase [42]. Then, these ions are transported into the cell by the high-affinity protein complex formed by the feroxidase FetC and the iron transporter permease FtrA [54]. The process of SIA in *A. fumigatus* is as follows: First, siderophores are secreted outside to chelate the iron in the external environment [35]. Then, iron ions are transported into the cells through the siderophore channel (Sit1/MirB) on the cell membrane; finally, the chelates are hydrolyzed by hydrolases (such as EstB), then Fe^2+^ is released [31]. The feroxidase *fetC*, the iron transporter permease *ftrA*, and several siderophore-synthesis-related genes in *A. cinnamomea* were successfully matched. Thus, we believe that RIA and SIA simultaneously exert effects on iron assimilation by *A. cinnamomea*.

The CWI signaling pathway, which is a conserved pathway in fungi, plays an important role in the growth and reproduction of fungi and the response to environmental stress [27]. The upstream of the CWI signaling pathway includes the baroreceptor WSC1, which is located on the cell membrane and is involved in cell wall reinforcement and repair in the responses to environmental stress [26,35]. In addition, the WSC1 receptor can respond to the concentration of iron ions [35,55]. In phytopathogenic fungi, the CWI signaling pathway is not only involved in the regulation of cell wall synthesis but also in the regulation of sporulation and pathogenicity [27]. The expression of the *bck1*, *mkk1*, and *slt2* genes in the CWI signaling pathway dramatically increased in the *A. cinnamomea* mycelial samples cultured with Fe^2+^ compared with that in the control (Figure 4). Therefore, in *A. cinnamomea*, the Wsc1 receptor on the cell membrane is believed to respond to the concentration of Fe^2+^ and ultimately promotes the expression of relevant genes in the CWI signaling pathway, thereby accelerating the cell wall synthesis and maturation of asexual spores.

In *A. fumigatus* and *A. nidulans*, two key transcription factors are involved in the regulation of cellular iron balance. They are the GATA-type zinc finger transcription factor SreA and the CCAAT-type bZip transcription factor HapX [42,43]. Both of the two factors can respond to the concentration of iron ions and transcriptionally inhibit each other [32,33]. When iron ions are sufficient in the environment, the expression of *sreA* is upregulated, which inhibits the transcription of *hapX* and the expression of genes related to iron ion assimilation, including the RIA and SIA pathways, and promotes the consumption of iron ions [32]. By contrast, when iron ions are deficient in the environment, then the expression of *hapX* is upregulated, which inhibits the transcription of *sreA* and promotes the expression of genes related to iron ion assimilation [33]. In the FluG-mediated sporulation signaling pathway in *A. cinnamomea*, FlbB, similar to HapX, is a bZip-type transcription factor, and BrlA, similar to SreA, is a transcription factor with two zinc finger structures. Moreover, the expression of the downstream genes of *flbB* and *brlA (flbd* and *abaA*) in *A. cinnamomea* mycelial samples cultured with Fe^2+^ had increased sharply compared with that in the control (Figure 4). Therefore, the target of HapX is speculated to be *flbd* and has the same function as FlbB, i.e., promoting the expression of *flbd*, and the target of SreA is *abaA* and has the same function as BrlA, i.e., promoting the expression of *abaA.*

Fur is also a regulatory factor that regulates the balance of intracellular iron ion concentration with multiple regulatory modes for the transcription of target genes. Fur has been reported to not only regulate the expression of genes related to iron ion transport but also to inhibit the expression of some other genes, such as *flbB* [56]. The typical regulation model for Fur in eukaryotes is as follows: When intracellular iron ions are sufficient, Fur combines with Fe^2+^, and the formed Fur–Fe^2+^ complex can bind to Fur–boxDNA in the upstream of the promoter to inhibit the expression of iron assimilation genes by inhibiting the binding of RNA polymerase. When intracellular iron ions are deficient, Fe^2+^ dissociates from Fur, leading to the reduction in the DNA-binding ability of Fur and activating the expression of the genes related to iron assimilation [38,39,54,55,56].

Nps2 is involved in the formation of spores in fungi. For example, the *Alternaria alternate* strain with the *nps2* deletion produces more spores earlier than the wild-type strain [57]. In the pathogenic bacterium *Cochliobolus heterostrophus*, *NPS4* plays an important role in virulence and conidia morphogenesis [40]. The *O*-methyltransferase TpcA can regulate the expression of *hapx* in *A. fumigatus* [58]. In *Ustilago maydis*, the deletion of the *urbs1* gene inhibits siderophore biosynthesis and places siderophore-mediated iron assimilation outside the reach of regulation [41].

**Figure 5:**
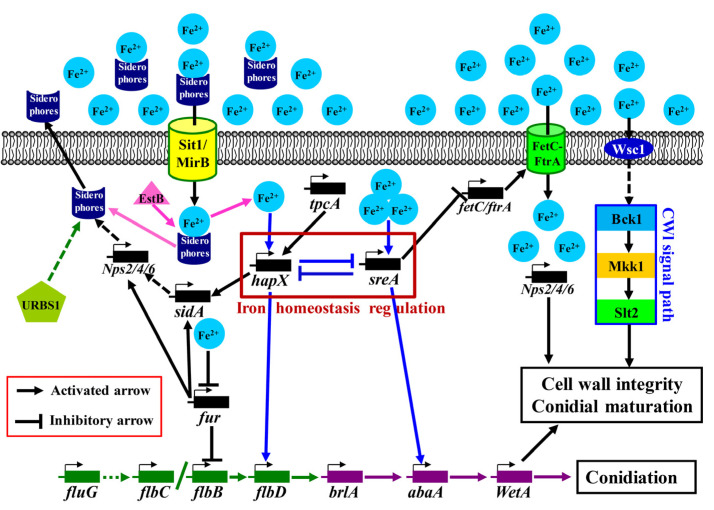
Model diagram of the Fe^2+^- and FluG-mediated signal pathway of the asexual sporulation of *A. cinnamomea* in submerged fermentation. Note: “green arrow”, upstream developmental activation pathway; “purple arrow”, central developmental pathway; “blue arrow”, iron homeostasis regulator pathway.

To sum up, the Fe^2+^- and FluG-mediated signal pathway of the asexual sporulation of *A. cinnamomea* was predicted (Figure 5) as follows: *A. cinnamomea* obtains iron ions through RIA and SIA. In RIA, ferrous iron ions are directly transported into cells by the high-affinity protein complex formed by a feroxidase (FetC) and an iron transporter permease (FtrA). In SIA, siderophores are secreted outside to chelate Fe^2+^ in the extracellular environment. Then, the chelates are transported into cells through the siderophore channels (Sit1/MirB) on the cell membrane and hydrolyzed by a hydrolase (EstB) in the cell to release Fe^2+^. The free siderophores can be extracellularly secreted again. Subsequently, HapX and SreA respond to the intercellular concentration of Fe^2+^. When iron ions are sufficient, the expression of *sreA* is upregulated, which inhibits the expression of *hapX*, *fetC*, and *ftrA*, thereby inhibiting iron assimilation. When iron ions are deficient, the expression of *hapX* is upregulated, which inhibits the expression of *sreA* and the consumption of iron. Furthermore, HapX and SreA affect *flbD* and *abaA*, respectively, and promote their expression.

The ferric uptake transcriptional regulator Fur can inhibit the expression of the *flbB* gene and further inhibit sporulation. However, when intercellular iron ion concentrations increase, Fe^2+^ combines with Fur to form the Fe^2+^–Fur complex to inhibit the expression of *fur* and alleviate the inhibition of *flbB*, which indirectly promotes sporulation. In addition, Fe^2+^ acts on the Wsc1 baroreceptor and promotes the expression of relevant genes in the CWI signaling pathway through a series of cascade reactions, thereby accelerating the cell wall synthesis and maturation of the asexual spores of *A. cinnamomea.* The *O*-methyltransferase TpcA can promote the expression of *hapX* then promote the synthesis of siderophores. The regulatory protein URBS1 can directly or indirectly regulate the synthesis of siderophores.

## 4. Conclusions

In the present study, comparative transcriptomics was used to reveal the molecular regulatory mechanisms underlying the asexual sporulation of *A. cinnamomea* promoted by Fe^2+^ in submerged fermentation. RIA and SIA were found to function in *A. cinnamomea*. In RIA, the ferroxidase FetC and the iron transporter permease FtrA directly transported Fe^2+^ into cells. In SIA, Fe^2+^ ions were transported into cells by siderophores through siderophore channels and released by the hydrolase EstB. Then, the transcription factors HapX and SreA responded to the concentration of Fe^2+^ and affected the expression of *flbD* and *abaA* in the FluG-mediated central signaling pathway, thereby promoting the sporulation of *A. cinnamomea* in submerged fermentation. However, the functions of relevant genes need to be further verified by more technical means, which is the focus and direction of the following research. Nevertheless, the present study contributes to improving the preparation efficiency of the inoculum (asexual spores) of *A. cinnamomea* in submerged fermentation, which saves production costs and improves production efficiency. For example, this method is easy to operate and inexpensive and is thus of great value for development and application. In addition, it provides a reference for further research on the molecular regulatory mechanisms of iron-ion-promoted asexual sporulation in other filamentous fungi.

## Figures and Tables

**Table 1:** Primers used for RT-qPCR.

Gene Name	Upstream Primer (5′→3′)	Downstream Primer (5′→3′)	Product (bp)
*flbD*	AATGTCTGAAGGTCGTGATGCC	GCCGTATCGTTAGCCGTATGG	126
*abaA*	TGTGCGAGTGCGGAGACC	GTAGACGACGGACAGGAGGAC	116
*bck1*	GTCAACAGTATAGATATGC	GTCAACAGTATAGATATGC	127
*mkk1*	CATAAAGGTCTTCGCTAT	CATAAAGGTCTTCGCTAT	165
*slt2*	ATCTCCTTTAGAAGACATC	ATCTCCTTTAGAAGACATC	103
18S rRNA	GCTGGTCGCTGGCTTCTTAG	CGCTGGCTCTGTCAGTGTAG	123

**Table 2:** Genes that may be related to the Fe^2+^-mediated asexual sporulation of *A. cinnamomea* among DEGs.

Unigene ID	Genome ID	Gene Name	Accession Number	*E* Value	Score
Cluster-140.3091	ACg001255	*mirB*	NC_007196.1	1 × 10^−19^	198
Cluster-140.1965	ACg005881	*ftrA*	NC_007198.1	5 × 10^−11^	82
Cluster-140.3564	ACg006970	*hapX*	NC_007198.1	4 × 10^−18^	183
Cluster-140.2153	ACg000929	*sreA*	NC_007198.1	6 × 10^−12^	89
Cluster-140.3137	ACg005708	*fetC*	NC_032094.1	2 × 10^−16^	138
Cluster-140.2788	ACg000854	*bck1*	NW_007930838.1	8 × 10^−16^	156
Cluster-140.3618	ACg001175	*uvt*	KJ158162.1	2 × 10^−17^	164
Cluster-140.2669	ACg007032	*urbs1*	NC_026479.1	7 × 10^−16^	154
Cluster-140.3500	ACg005433	*sit*	MF447899.1	6 × 10^−14^	102
Cluster-140.2357	ACg001741	*fre*	NC_007197.1	4 × 10^−14^	100
Cluster-140.4088	ACg006852	*slt2*	AEU60018.1	2 × 10^−90^	326
Cluster-140.2081	ACg007003	*ssiG* 06045	XP_001593123.1	6 × 10^−18^	189
Cluster-140.1623	ACg002353	*feoB*	NC_000913.3	3 × 10^−16^	145
Cluster-140.3137	ACg005708	*tpcA*	NW_020939752.1	1 × 10^−18^	175
Cluster-140.3451	ACg003216	*nrps*	KIM81356.1	0	2771
-	ACg008442	*nps2*	NC_031953.1	7 × 10^−11^	84
Cluster-140.3206	ACg002074	*nps4*	KY471559.1	1 × 10^−25^	268
Cluster-140.4587	ACg007029	*clpP*	NC_000964.3	3 × 10^−15^	126
Cluster-140.4385	ACg003470	*mkk1*	NW_007930837.1	2 × 10^−10^	79
-	ACg000676	*sidA*	NC_007194.1	8 × 10^−19^	202
Cluster-140.3564	ACg006969	*yvmB*	NC_020507.1	2 × 10^−18^	179
Cluster-140.132	ACg001175	*fur*	NC_016845.1	3 × 10^−14^	97
Cluster-140.2003	ACg007734	*estB*	NC_007196.1	5 × 10^−16^	149
Cluster-140.2389	ACg007303	*wsc1*	NC_007198.1	3 × 10^−16^	146

Note: “Unigene ID” is the code of the unigene generated in the process of software assembly; “Genome ID” is the code corresponding to the gene matched with the unigene in the *A. cinnamomea* genome (ACg); “-” indicates an unmatched gene in the *A. cinnamomea* genome database; “accession number” is the NCBI number of the protein matched with a unigene in the local protein database; “*E* value”, “Homology”, and “Coverage” are used to describe the matching between a unigene and the corresponding protein in the local protein database. If the *E* value is low, then homology and coverage are high, and the matching degree is high. It was considered as a successful match when the *E* value ≤ 10^−6^.

## Data Availability

Data are contained within the article or Supplementary Materials.

## Supplementary Materials

The following supporting information can be downloaded at: https://www.mdpi.com/article/10.3390/jof9020235/s1, Table S1: The unigene database of *Antrodia cinnamome* by RNA-seq; Table S2: The FPKM values of the unigenes from RNA-seq; Table S3: The obtained differentially expressed genes (DEGs) of enrichment analyses. Table S4: The DEGs from pairwise comparison and their functioin annotations.
